# Internet of things issues related to psychiatry

**DOI:** 10.1186/s40345-020-00216-y

**Published:** 2021-04-02

**Authors:** Scott Monteith, Tasha Glenn, John Geddes, Emanuel Severus, Peter C. Whybrow, Michael Bauer

**Affiliations:** 1 Michigan State University College of Human Medicine, Traverse City Campus, Traverse City, MI USA; 2 ChronoRecord Association, Fullerton, CA USA; 3 Department of Psychiatry, University of Oxford, Warneford Hospital, Oxford, UK; 4 Department of Psychiatry and Psychotherapy, University Hospital Carl Gustav Carus Medical Faculty, Technische Universität Dresden, Fetscherstr. 74, 01307 Dresden, Germany; 5 Department of Psychiatry and Biobehavioral Sciences, Semel Institute for Neuroscience and Human Behavior, University of California Los Angeles (UCLA), Los Angeles, CA USA

**Keywords:** Internet of things, Psychiatry, Security, Privacy, Bipolar disorder

## Abstract

**Background:**

Internet of Things (IoT) devices for remote monitoring, diagnosis, and treatment are widely viewed as an important future direction for medicine, including for bipolar disorder and other mental illness. The number of smart, connected devices is expanding rapidly. IoT devices are being introduced in all aspects of everyday life, including devices in the home and wearables on the body. IoT devices are increasingly used in psychiatric research, and in the future may help to detect emotional reactions, mood states, stress, and cognitive abilities. This narrative review discusses some of the important fundamental issues related to the rapid growth of IoT devices.

**Main body:**

Articles were searched between December 2019 and February 2020. Topics discussed include background on the growth of IoT, the security, safety and privacy issues related to IoT devices, and the new roles in the IoT economy for manufacturers, patients, and healthcare organizations.

**Conclusions:**

The use of IoT devices will increase throughout psychiatry. The scale, complexity and passive nature of data collection with IoT devices presents unique challenges related to security, privacy and personal safety. While the IoT offers many potential benefits, there are risks associated with IoT devices, and from the connectivity between patients, healthcare providers, and device makers. Security, privacy and personal safety issues related to IoT devices are changing the roles of manufacturers, patients, physicians and healthcare IT organizations. Effective and safe use of IoT devices in psychiatry requires an understanding of these changes.

## Background

The era of the Internet of Things (IoT) has arrived, where smart, connected technologies are being embedded in everyday objects such as cars, toothbrushes, washing machines, and physical infrastructure on a massive scale. The use of IoT devices for remote monitoring, diagnosis and treatment, is viewed as an important way to improve and expand individualized medical care and assist with lowering costs, including for bipolar disorder and other mental illness (Deloitte 2018; de la Torre Díez et al. 2018). While there is no standard definition, the IoT describes “the extension of network connectivity and computing capability to objects, devices, sensors and items not ordinarily considered to be computers” (Internet Society 2015). IoT devices can be thought of as physical devices with embedded technology that can sense, generate, store, and send data, and sometimes respond to commands via actuators that can modify the physical world. Increasingly, IoT devices will be installed in the home for medical purposes as selected by patients or recommended by physicians.

Today, a diverse range of IoT devices are found in homes, retail businesses, public spaces, hospitals and healthcare facilities, vehicles, utility infrastructure, and are directly worn by consumers. Virtually every consumer electronics device is now sold as a connected IoT device (NIST 2019). The scale of the IoT is unprecedented, with estimates of 30 billion connected devices by 2020 (Nordrum 2016), and that half the total global Internet traffic will be machine-to-machine connections by 2022 (Cisco 2019). About 71% of homes in North America, and 57% in Western Europe have at least one IoT device (Kumat et al. 2019). The scale, complexity and passive nature of data collection creates many new and unique challenges for the use of IoT devices in psychiatry, with functions for detection of emotion, mood state, stress, activity patterns and cognitive skills (Glenn and Monteith 2014; Abdullah and Choudhury 2018; APA 2019). This paper will discuss IoT issues related to psychiatry and general medicine with examples for bipolar disorder, including the major challenges to security, safety and privacy, and the complex impacts on manufacturers of IoT devices, patient users, and healthcare organizations.

### IoT background

A confluence of factors led to the rapid increase in IoT devices (Internet Society 2015; GAO 2017). The expansion and decreasing costs of multiple types of networks (e.g. broadband, cellular, and short range wireless networks including Wi-Fi, Bluetooth, Zigbee) led to near ubiquitous connectivity. Inexpensive miniaturization of electronics enabled the development of parts, such as sensors, that fit in very small objects, including biosensors for healthcare monitoring (Kim et al. 2019). Cloud computing allowed distributed IoT devices to interact with back-end processing centers for data management and storage. New data analytic techniques allowed aggregation and analysis of the large volumes of data created by IoT devices. The fundamental Internet Protocol (IPv6) was updated to vastly increase the number of available network addresses. Finally, new business models were developed for the IoT, based on data collection.

A typical home network consists of a wireless router connected to the Internet. IoT devices are connected to the wireless router either directly or indirectly through a hub device. Although a smartphone or tablet app may be used to initially configure the IoT device, the data collected by the IoT device are sent using the wireless router to a server at the manufacturer or IoT service provider. An IoT device for home use contains electronics for data collection, often involving sensors, cameras, and microphones. Some IoT devices can subsequently be managed by a smartphone app or website. Examples of the variety of IoT devices available for home, consumer health and fitness, and approved medical IoT devices are shown in Table 1.

**Table 1:** Examples of IoT devices

Home devices:
Automobile systems
Bathroom appliances
Door and window locks
Kitchen appliances (refrigerators, stoves)
Lighting
Security cameras
Smoke alarms
Speakers
Thermostats
Toys
TVs
Utility meters
Vacuum cleaners
Voice assistants
Consumer health/medical devices:
Baby clothes that monitor respiration
Electronic pill bottles
Environmental chemical sensors
Fitness trackers
Football helmets that analyze impacts
Scales
Sleep monitors
Smart toothbrush
Thermometers
Video games to improve attention
Voice assistants to refill prescriptions
Water bottles
Wearable blood pressure monitors
Wearable ECG monitors
Wearable sweat sensors
Approved medical devices from physicians:
Cardiac implanted devices (pacemakers, defibrillators)
Cochlear implant
Drug delivery systems
Foot drop implants
Glucose monitors
Implanted biosensors
Ingestible medications
Neurostimulators
Oxygen saturation
Patient identification and tracking
Smart inhalers
Vital sign monitors

In psychiatric research, IoT devices are often wearables, such as wristwear, clothing, belts and body patches, containing sensors to measure physical activity and heart rate variability. The sensor data from the wearables may be combined with other data sources, and used to classify emotional reactions, mood states and stress in various psychiatric disorders (Reinersten and Clifford 2018; Zhu et al. 2019). Examples of research involving IoT devices and bipolar disorder are shown in Table 2, with studies using activity patterns to distinguish bipolar disorder from other diagnoses, and heart rate variability to predict mood state.

**Table 2:** Example studies of patients with bipolar disorder using data from IoT devices (wearable devices and ingestible sensors)

Study	IoT device	Measure	Participants	Study aim
Tanaka (2018)	Wrist-worn accelerometer	Physical activity	94 inpatients: 57 with MDD; 35 BP with depression.*	Distinguish activity patterns between adults with BP and MDD
Faedda (2016)	Wrist-worn accelerometer	Physical activity	155 youths: 48 with BP, 44 with ADHD; 21 with ADHD + MDD; 42 controls	Distinguish children with BP from those with ADHD and healthy controls
McGowan (2020)	Wrist-worn accelerometer	Physical activity	87 patients: 31 with BP; 21 with BPD; 35 healthy controls	Compare rest-activity patterns in those with BP, BPD, and healthy controls
Merikangas (2019)	Wrist-worn accelerometer	Physical activity	242 adults: 54 with BP; 91 with MDD; 97 healthy controls	Compare associations between activity, sleep, energy and mood in those with and without mood disorders
Rodríguez-Ruiz (2020)	Wrist-worn accelerometer	Physical activity	55 patients: 23 with BP or MDD; 32 healthy controls	Compare activity in the day and night to classify depressive episodes
Janney (2014)	Elasticized belt containing an accelerometer	Physical activity	60 patients: 41 with BPI, 17 with BPII; 2 with BP NOS	Understand the physical activity and sedentary behavior of adults with BP
Kappeler-Setz (2013)	Socks with sensor of skin conductance	Electrodermal activity (changes in sweat gland activity)	Eight healthy subjects; feasibility study	Use for long-term monitoring of patients with BP
Valenza (2014)	T-shirt embedded with electrodes and sensors	ECG	Eight patients with BP	Predict mood states from heart rate variability in patients with BP
Nardelli (2017)	T-shirt embedded with electrodes and sensors	ECG	Eight patients; six with BPI; 2 with BPII	Study of diurnal and nocturnal heartbeat dynamics in BP mood states
Gentili (2017)	T-shirt embedded with electrodes and sensors	ECG	Eight patients with BP	Predict mood changes from heart rate dynamics in patients with BP
Kopelowicz (2017)	Ingestible sensor in tablets	Ingestion of Abilify MyCite (aripiprazole)	49 patients; 22 with BPI; 15 with schizophrenia; 12 with MDD	Implement a call center to facilitate adherence monitoring of patients using a digital pill system

*BP bipolar disorder, *MDD* major depressive disorder, *ADHD* attention deficit/hyperactivity disorder, *BPD* borderline personality disorder

### Security challenges

There are security challenges with IoT devices that differ from those involving traditional computers. Many IoT devices are battery powered, and have severe constraints on power, memory, and processing resources. These devices lack the capacity to run conventional operating systems, and to support encryption or anti-virus software (IoT Cybersecurity Alliance 2017; Bacceli et al. 2013). Many IoT devices lack a software upgrade process, or have only a very cumbersome process to upgrade (GAO 2017). IoT devices that are embedded in products or systems may be inaccessible. Many IoT devices are never rebooted, have a service life much longer than for traditional computer equipment, and could contain obsolete or dangerous hardware and software (Intel 2016). A poorly secured IoT device may potentially affect the security of every interconnected device, local and remote (Internet Society 2015). This allows hackers to target nontraditional devices such as a television or refrigerator, both to exploit home networks and launch an external cyberattack (NSA 2016). Collecting data using cloud computing also presents many potential opportunities for data mismanagement and improper security controls (GAO 2017).

Many FDA-approved medical devices have a long life span and were developed before the era of interconnectivity and the need for cybersecurity (Schwartz et al. 2018). Most digital devices approved by the FDA would today be considered IoT devices. The FDA now recommends monitoring cybersecurity throughout the entire product life-cycle (FDA 2016a). If cybersecurity issues require a software or firmware update, the device manufacturer is responsible for updates to address the cybersecurity risk (FDA 2020a). Changes solely to strengthen cybersecurity typically do not need FDA review and should be performed routinely (FDA 2016a, b, 2020a), but implementation is often delayed with so many diverse stakeholders (Woods et al. 2019). However, if the software or firmware changes affect the device safety or effectiveness, FDA approval is required prerelease (FDA 2016a). The FDA has adopted a premarket submission standard to demonstrate steps taken to mitigate cybersecurity risks (UL 2018), requires a unique device identifier (FDA 2019a) and has plans to adopt other measures to improve medical device safety (FDA 2018). Cybersecurity is an international problem and starting in 2020, new European Union Medical Devices Regulation will tighten regulatory controls, increase device traceability throughout the supply chain, and require ongoing post-market surveillance (McDonough 2019).

### Safety challenges

Some medical IoT devices have the potential to directly endanger the safety of the owners (GAO 2017). Safety and security concerns of IoT devices are interconnected, as poor security impacts safety and safety violations may impact security (Zalewski et al. 2019) Although the FDA has no confirmed reports of patient harm due to a cybersecurity incident involving a medical device (FDA 2020a), the FDA has released 11 safety warnings since 2013 involving insulin pumps, implanted cardiac devices, cardiac monitors, infusion pumps and central patient monitoring displays (FDA 2020a). In 2020, the FDA identified 12 cybersecurity vulnerabilities with Bluetooth Low Energy wireless technology, a communications protocol used in medical devices from several manufacturers (FDA 2020b; DHS 2020). While patients want to be told of cybersecurity risks with medical devices (FDA 2019b), impacted patients and clinicians may react conservatively. In a study of a firmware update to mitigate a cybersecurity vulnerability found in an implanted cardiac pacemaker, only about 25% of those affected chose to upgrade (Saxon et al. 2018). Other technology issues may lead to safety risks with medical devices. For example, although a continuous glucose monitor was functioning properly, a server outage at the manufacturer stopped alerts and other communications to parents and caregivers (Parmer 2019). There may also be safety risks from consumer IoT health and fitness devices. For example, the close proximity of some wearables to the body may lead to skin irritations from chemicals in the device, and chemical burns from battery leaks (CPSC 2017).

### Privacy challenges

The use of IoT devices in the home, and of wearables, encroaches on spaces long considered and valued as private—the home and the body (Rosner and Kenneally 2019). IoT devices are eroding the boundaries between public and private, and create the potential for continuous monitoring of activities, speech, behavior and emotions (Internet Society 2019). People may no longer be able to keep privacy boundaries in place. However, privacy remains very important to most. In a 2019 survey, more than 80% of Americans found the potential risks outweigh the benefits when companies collect data, and felt they had very little or no control over the data collected by companies or the government (Pew Research 2019; Auxier and Rainie 2019). In a survey of consumers in five countries, 75% distrust the way that data are being shared (Internet Society 2019). Nearly constant surveillance may lead to chilling and conforming effects on behavior in the home (Rosner and Kenneally 2019; Oulasvirta et al. 2012; Kamiinski 2014). Privacy is a particularly important concern for individuals with psychiatric disorders, especially due to the stigma (Monteith and Glenn 2016; Bauer et al. 2017).

Many consumers may not be aware that “surveillance capitalism” is now the business model in virtually every economic sector, including every smart product or personalized service (Zuboff 2019). Digitized human experience is now raw material for translation into behavioral predictions. Massive amounts of data from all possible digital activities (online, smartphone, financial, IoT devices at home including health tracking and monitoring, urban and commercial IoT) are collected. These data are then combined, analyzed and packaged as “prediction products” to tell business customers how people will behave now and in the future (Zuboff 2019). People with mental illness may be especially at risk of harm from errors and biases in data and algorithms associated with automated decision making (Monteith and Glenn 2016; Bauer et al. 2017).

The fundamental approach to privacy on the Internet is based on notice and choice with the user providing consent to a privacy policy. However, most IoT devices have no means for user interaction such as a screen, mouse or keyboard (Peppet 2014). IoT device privacy policies are often on a web site, and do not clarify the ownership, use and sale of all collected data (Peppet 2014). Consumers may not realize that data from health and fitness trackers may be routinely sent to third parties, or even that their IoT devices are interacting with the Internet. Some individuals may provide consent for data collection without understanding the scope, such as with an IoT enabled television that includes voice recognition (GAO 2017). A simple binary consent may not be sufficiently flexible for the online environment (International Institute of Communications 2012). Furthermore, many users routinely ignore or do not carefully read online privacy policies (Pew Research 2019; West 2019).

The use of prescribed medical digital devices creates new challenges related to consent. In addition to traditional medical consent based on discussion with a physician, the patient often has to register with the company who manufactured the device and provide consent to a user agreement (Klugman et al. 2018). Corporate user agreements are often long, written in legalese, and are non-negotiable. Yet mental illness may interfere with the capacity to provide traditional informed consent (Okai et al. 2007; Lepping et al. 2015; Morán-Sánchez et al. 2016). Other privacy issues associated with prescribed medical devices relate to data ownership, data use, and data sharing by device manufacturers. Health related privacy remains very important to patients. In a 2019 study of 4000 adults representative of the US population, only 10% want to share health data with technology companies (Rock Health and Stanford 2019). Another concern is that consumers may not understand that de-identified data are routinely vulnerable to re-identification techniques in the era of big data (Narayanan et al. 2016; Rocher et al. 2019). For example, in a dataset from 14,451 individuals with protected health data removed, 95% of adults were reidentified using aggregated physical activity data measured by accelerometers (Na et al. 2018).

## New roles in the IoT economy

### New roles for manufacturers

Embedded processors are being added to everyday objects, yet most traditional manufacturers lack in-house technical expertise and are unaware of security risks and interoperability issues (Sadler 2017; Hypponen and Nyman 2017). In the highly competitive, global consumer products market, manufacturers rush to get a device to market, focus on lowering costs and gaining market share, and often release products with little testing (Sadler 2017). The primary source of recurring revenue for most IoT devices is not selling multiple devices to the same customer, but selling the data collected by the devices (Anderson 2018). Manufacturers rely on third-party support for product design, component purchase, and assembly, with hardware and software components frequently re-used in IoT products beyond what they were initially designed for (GAO 2017; Sadler 2017). The use of identical or near-identical software and firmware in many devices can magnify the impact of a successful attack when a vulnerability is found, and increases the potential for successful attacks (GAO 2017; Intel 2016). The complex global supply chain also poses diverse security risks (Kshetri and Voas 2019; Radanliev et al. 2019).

The result is that security built into IoT devices is far weaker than in traditional devices on the Internet, such that IoT devices are now a larger target for hackers than traditional web applications and servers (Boddy et al. 2018). For example, the public and private keys that are used in certificates to ensure encryption security can be compromised if random number generation is flawed. In a study of 75 million RSA certificates from the Internet, keys shared a common factor based on a random number in 1 of 172 certificates from IoT devices versus 1 in 20 million from standard websites (Kilgallin 2019). These weak keys expose users to a wide variety of potential harms. A hacker with a re-derived private key for a SSL/TLS server certificate may impersonate a server, capture login credentials, medical and financial data, decrypt stored communications, and intentionally cause a device to malfunction (Kilgallin 2019). Another example relates to the apps that accompany many IoT devices. In a study of apps that accompany 96 popular IoT devices (32 apps), 31% had no encryption, and another 19% had poor encryption (Mauro et al. 2019). IoT startups may introduce a product but quickly go out of business or abandon a device, but the device may remain in a home for many years without any potential for security upgrades (Fu et al. 2017).

In 2020, a new law in the UK requires manufacturers to provide unique passwords for individual IoT devices that are not resettable to universal factory settings, state the minimum length of time they will provide security updates, and provide a public contact point to report vulnerabilities (Gov.UK 2020). This is an important step towards improving IoT security and protecting consumers.

### New roles for patients

For healthcare, patients will use a combination of consumer health and fitness IoT devices and prescribed medical IoT devices. Consumer IoT devices provide insufficient security information in their manuals or websites (Blythe et al. 2019), and patients often get security advice from family and friends (Redmiles et al. 2016). In a study of 1878 websites providing security advice, only 25% were written at a standard reading level (e.g., Reader’s Digest) with the rest harder to understand (Redmiles et al. 2018). Patients will not only be the user but will install, configure, manage and decommission consumer IoT devices, and prescribed IoT medical devices that communicate with the provider. Patients may not realize that ongoing maintenance may be required for a medical device including software or firmware updates, battery changes, and sensor replacements (Woods et al. 2019; Klugman et al. 2018). Some routine behaviors may negate the validity of data collected from IoT devices and trigger serious privacy and security concerns. When consumers buy a new smart device, they focus on features and functions and overlook security settings (NSA 2016). In multiple surveys in the US, Canada and the UK, the majority of consumers did not change their router’s default password (Powell 2018; De Leon 2019; ESET 2019). When consumers borrow, rent, gift or resell their used IoT devices without removing their association to the device, collected data may be assigned to the wrong individual (Khan et al. 2018). Patients with mental illness may have fewer digital skills than the general public (Bauer et al. 2017, 2020).

Patients may lack the knowledge to follow security advice. For example, the FBI recommends that devices with private and sensitive data, such as a laptop or medical device, be kept on a separate home network from other IoT devices such as a refrigerator (FBI 2019). However, a patient's medical devices are usually located on the same wireless network as all the home IoT devices from many manufacturers (Fu et al. 2017). The result is the safety and security vulnerabilities of home and provider systems are combined, with each becoming a potential backdoor vulnerability to the other (Fu et al. 2017). Patient medical devices that are connected to medical facilities pose a major cybersecurity threat and are often viewed as the weakest link within healthcare networks (Deloitte 2018; Sun et al. 2019; Grau 2020). In addition to many security issues in a wide range of home IoT devices, a 2019 US study found that many wireless routers for home networks lack basic security protections (De Leon 2019).

### New roles for healthcare organizations

Healthcare organizations must recognize the increased risks associated with interconnected medical devices and take an aggressive role to protect patients, physicians, and staff, and medical data from cybersecurity threats. This protection must extend to the rapidly growing number of remote connections from patients at home transmitting large volumes of data from medical devices or health and fitness devices. In 2017, the US Cybersecurity Task Force rated healthcare cybersecurity in “critical” condition (HHS 2017), and for 2019, ECRI Institute found cybersecurity attacks from hackers exploiting remote access as the number one health technology hazard (ECRI 2018). Every aspect of the interconnected healthcare network, including users of all backgrounds, hardware, firmware, software and communications channels, present different levels of risk and are part of the security problem (ECRI 2018). Providing adequate security protection in healthcare is resource intensive and will require considerable investment to improve IT security skills, communicate and coordinate with device manufacturers and patients, implement ongoing, comprehensive, multi-layered security controls, and deploy measures to promptly address vulnerabilities and install updates (HHS 2018). Healthcare IT organizations should take the lead in establishing ongoing IoT related education for all physicians, staff, and connected patients, including for the busy, disinterested, compromised or financially challenged.

### Limitations

There are many limitations to this paper. The specific benefits, efficacy, and risks of IoT devices used in psychiatry were not discussed, including technology concerns such as sensor accuracy, manufacturing practices such as sensor and part substitutions across the product life cycle, and the use of proprietary algorithms (Bauer et al. 2020). Proposed new approaches to validation and efficacy testing (Coravos et al. 2020), and discussion of the FDA Digital Health Software Pre-certification Program were omitted (Lee and Kesselheim 2018). The potential conflict of interest for clinicians collaborating with technology companies on the development of IoT devices was not discussed.

Proposed technical standards, government regulations, and commercial and academic approaches to improve privacy and security of the IoT were not included. Technical details related to interoperability of data from diverse devices and systems, software quality, data quality, operations, bandwidth, edge processing outside the data center, and cloud computing were omitted. Privacy challenges related to 5G cellular networks were not included (Marcos 2017). Details regarding cybersecurity and safety issues for regulated medical devices were not provided. Unique challenges of some medical devices, such as the need for quick and simple access in emergencies, were not discussed (Sametinger et al. 2015). Methods to increase physician and patient knowledge of the IoT, legal and ethical issues including provider and manufacturer responsibility for errors, and contractual issues were not included. Digital inequalities, including equitable access to IoT devices, and differences in patient skills, and the impacts of security or privacy breaches on patient trust of physicians and healthcare organizations were not discussed. The environmental issues of energy consumption and carbon footprint for the billions of IoT devices and systems used to analyze the collected data were not discussed (Bol et al. 2015; Ashrad et al. 2017).

The article search occurred between December 2019 and February 2020. Since the pandemic began, the growth rate of new IoT devices has slowed due to lower consumer and enterprise demand, manufacturing shutdowns, supply chain interruptions, and reduced project funding (GSMA 2020; ABI Research 2020). Despite this, the use of some healthcare IoT devices such as digital thermometers is growing (Leuth 2020), and recovery of the IoT marketplace is expected to start in 2021 (GSMA 2020).

## Conclusions

It is inevitable that more IoT devices are coming to psychiatry In the future, there will be a choice of IoT medical devices for psychiatrists to recommend including for bipolar disorder. Patients will increasingly use IoT medical devices to monitor general medical conditions, in addition to consumer health and fitness devices. While IoT devices offer many potential benefits for remote monitoring and treatment, there are risks associated with IoT devices, and from the connectivity between patients, healthcare providers, and device makers. Understanding these risks is necessary for optimal use of IoT devices in psychiatry. Security, safety and privacy issues are changing the roles of manufacturers, patients and healthcare IT organizations. It is important to determine how these devices can be used in real-world settings, to obtain data that are clinically valuable, and to avoid security, privacy and safety issues for the patient, physician and healthcare organization.
